# Bilateral Thalamic Stroke Arising From an Occlusion of the Artery of Percheron: Barriers to Diagnosis, Management, and Recovery

**DOI:** 10.7759/cureus.19783

**Published:** 2021-11-21

**Authors:** Alexander M Satei, Chaudhary A Rehman, Sunil Munshi

**Affiliations:** 1 Department of Stroke, University of Nottingham, Nottingham, GBR; 2 Department of Diagnostic Radiology, St. Joseph Mercy Oakland Hospital, Pontiac, USA; 3 Department of Stroke, Nottingham University Hospitals NHS Trust, Nottingham, GBR

**Keywords:** bilateral thalamic infarct, bilateral stroke, midbrain, thalamus, artery of percheron

## Abstract

A 90-year-old male patient presented with excessive somnolence, right-sided weakness, and left facial droop. CT and MRI scans of the head, taken several days after initial head CT proved to be non-revealing, demonstrated a bilateral thalamic stroke, a rare phenomenon. The infarct arose in the territory of the artery of Percheron, an anatomic variant in which a single artery supplies both sides of the thalamus and midbrain. When this artery becomes occluded, it results in severely dysregulated consciousness and alertness. This type of stroke proved challenging for the medical team, due to poor resolution of initial imaging, as well as the therapy teams, due to the constant need for sleep. This case report outlines how barriers in diagnosis and management make knowledge of the artery of Percheron and its occlusion crucial to patient care and recovery.

## Introduction

The artery of Percheron is a rare anatomic variant that provides a means by which a bilateral thalamic stroke can occur. It is a single branch from the posterior cerebral artery which supplies both sides of the thalamus and midbrain [1]. When infarcted, patients present with unique features such as excessive drowsiness and confusion [1]. Understanding its anatomy, the structures it supplies, and the presentation of a patient affected by a stroke in this area helps in identifying and understanding affected patients. 

There are three major difficulties with this type of stroke. The first arises from somnolence, resulting in poor response to therapy and problems soliciting a history from the patient, the second being the poor sensitivity of early imaging, and the third being a lack of knowledge of its anatomy and the effects of its occlusion. Due to the fast-paced environment in which stroke care needs to be delivered, these three barriers to care can have a large impact on a patient's prognosis.

## Case presentation

A 90-year-old male was found in his home slumped to his right side and unable to be awakened. Paramedics calculated an 8/15 Glasgow Coma Scale (GCS) score, which remained the same on admission. On presentation to the hospital, the patient had left facial droop, bilateral pinpoint pupils, and right-sided weakness. The National Institutes of Health Stroke Scale (NIHSS) score on admission was 26, suggesting a severe stroke. All other observations on admission were non-revealing, including vital signs, biochemical tests, and systems review.

The patient’s neurological medical history included two previous transient ischemic attacks and suspected dementia. Other past medical history included chronic obstructive pulmonary disease, hypertension, abdominal aortic aneurysm repair, stage 3 chronic kidney disease, adult polycystic kidney disease, hypercholesterolemia, and aortic valve sclerosis. He was an ex-smoker of unknown pack-years and prior to admission was living independently.

Initial head CT performed only showed chronic small vessel disease with periventricular leukoaraiosis, consistent with his age. Thrombolysis was initiated with an intravenous tissue-plasminogen activator, however, the patient’s GCS declined to 6/15 approximately 40 minutes after the start of thrombolysis. A repeat head CT was performed, again showing no acute findings. To prevent further deterioration, a decision to monitor the patient’s condition conservatively was made. 

Over the next few days, the patient remained very somnolent. When the patient was able to awaken, he was only capable of responding to simple commands. Speech and language therapists noted that fatigue was limiting conversations, with the patient speaking with imprecise articulation and at a low volume. Occupational therapists concurred that the patient was poorly engaged during sessions, while physiotherapists classified this patient as having limited rehabilitation potential.

**Figure 1:**
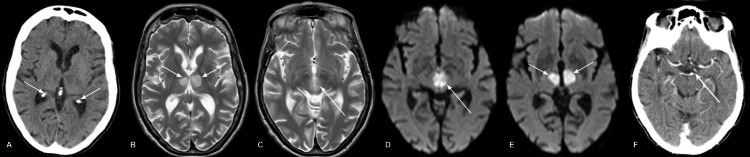
CT and MR imaging of the patient. Third CT scan showing hypoattenuation in both thalami (A). MRI-T2 showing bilateral thalamic infarcts (B) and midbrain infarcts (C). DWI-MRI showing bilateral midbrain infarcts (D and E). MR angiogram showing occlusion of the left posterior cerebral artery supplying the artery of Percheron (F). CT: computed tomography; MRI: magnetic resonance imaging; DWI: diffusion-weighted imaging

Stroke was finally confirmed upon a third head CT several days after admission, which showed bilateral thalamic infarcts. Other findings included hyperdensity at the basilar tip and left posterior cerebral artery, likely due to wall calcification of vessels. An MRI revealed bilateral hyperintensities in the thalami, consistent with acute bilateral thalamic stroke, with small left parietal cortical infarction (Figure 1).

Imaging also confirmed the arterial location of the infarction, a single branch of the pre-communicating (P1) segment of the left posterior cerebral artery known as the artery of Percheron (AOP). Over the next month, the patient participated in multidisciplinary rehabilitation sessions. He had a gradual return of physical function and with it, his sense of humor returned, much to the delight of his family and the attending staff. He was unable to care for himself independently and was eventually discharged to a care home. Six months later, he was mobile with a walking stick and had a reasonable quality of life. One year after the initial presentation to the hospital, he passed away from an intercurrent chest infection.

## Discussion

In most cases, the thalamus benefits from bilateral overlapping arterial supply from the perforating branches of the posterior cerebral artery and posterior communicating artery [2]. This vascular supply is categorized into tuberothalamic, paramedian (thalamosubthalamic), inferolateral, and posterior choroidal territories [3]. Furthermore, this supply varies individually. For example, in one-third of the population, the tuberothalamic artery is absent, with its territory instead supplied by the paramedian artery [4]. This underlies the basis of the rare nature of bilateral thalamic strokes; such strokes only represented 0.6% of all ischemic strokes in a study involving 2750 patients [5]. When a bithalamic stroke is suspected, an AOP infarct is considered the main presumptive diagnosis until proven radiologically [6].

**Figure 2:**
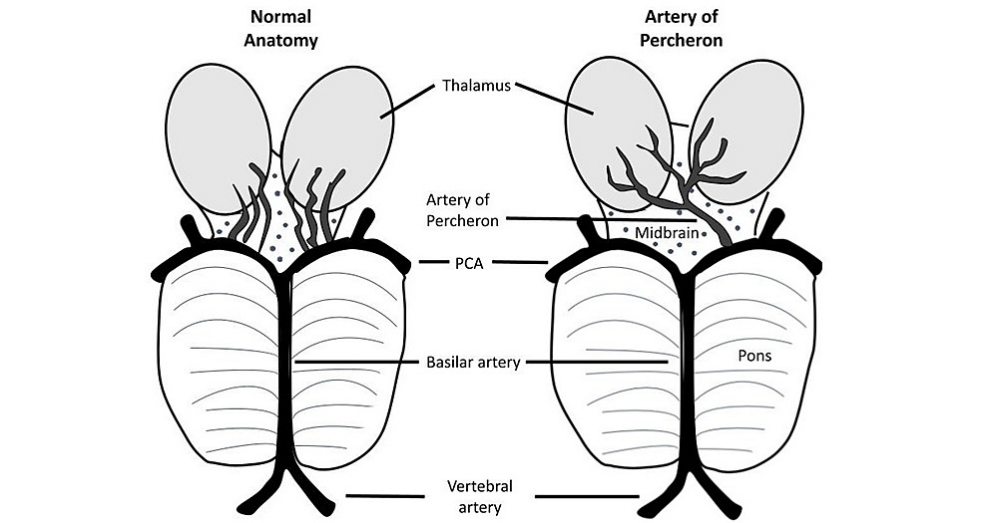
Diagrammatic representation of the normal anatomy compared to the artery of Percheron. PCA: posterior cerebral artery The image is created by the authors of this study.

The prevalence of the AOP in the general population is not clear, with estimates ranging from 11.7% of cadavers in one study [7], and up to 33% in another [8]. An AOP infarction represents 0.1-2% of all ischemic strokes [9] and carries a 12% mortality rate [1]. It can be indicated by a V-shaped hyperintensity in the midbrain on imaging, known as the “V sign,” found in 67% of cases; this sign was not seen in this case [9]. In patients with an AOP infarction, associated strokes may be found in the cerebellum (19%), occipital lobe (11%), and middle cerebral artery (14%) [10]. In this case, a simultaneous, small infarction was seen in the left parietal lobe. Figure 2 shows a diagrammatic representation of the AOP.

There are four main acute features of an AOP infarct. These include vertical gaze palsy (65%), impaired memory (58%), confusion (53%), and coma (42%) [11]. This case presented with two of these features - confusion and sleepiness. Long-term consequences of a stroke in the AOP include orientation and concentration deficits, behavioral disturbances, and impaired memory [9].

There are also four patterns of ischemic damage due to an AOP stroke. These include involvement of bilateral paramedian thalamus and the midbrain (43%), bilateral paramedian thalamus without the midbrain (38%), bilateral paramedian thalamus, anterior thalamus, and midbrain (14%), and bilateral paramedian and anterior thalamus without midbrain (5%) [9]. Imaging in this case demonstrated the first pattern of ischemia. Features of added midbrain involvement could include hemiplegia, ataxia and other movement pathology, and oculomotor disturbances [12]. This case involved right-sided weakness and visual inattention.

The AOP stroke remains an elusive obstacle for the medical community to tackle for several reasons. The first hurdle relates to the state of heavy somnolence. In addition to the difficulties created for therapy teams already discussed, for cases involving unknown patients or those with no next of kin, taking an initial history could become impossible. In this case, the patient presented with a well-recorded past medical history, as well as family members who were available for questioning at the time of admission.

The second handicap arises from a radiology perspective. In most cases, initial cerebral CT shows no acute findings. This can mislead the medical team to begin a search for an alternative diagnosis, despite the overwhelming evidence of stroke features or thalamic dysregulation. In a different case study on AOP infarction, 24 days passed after admission before a second head CT was performed, which at that point showed ischemia in both thalami and the pons [13]. During this time, the only treatment provided was intubation, and the patient passed away shortly after. In this case, head CT was more rapidly performed, as the medical team had suspected its possibility. As a result, the AOP stroke often becomes a clinical diagnosis based on symptoms, and its prognosis depends on the judgment of the team in charge.

It is therefore unfortunate that the third barrier involves the lack of knowledge of the AOP and the features of its pathology. In this case, the physicians treating the patient initially were even considering putting him on the end-of-life pathway without realizing that he would wake up in a few days. With improved comprehension by means of further exposure to patients suffering from its pathology, as well as research and reviews on the topic, medical teams will feel more confident in its diagnosis and care. Until then, the AOP will remain home to the elusive “sleep stroke,” with frequent incorrect therapy and diagnoses.

Like most stroke cases, recovery is dependent on the patient’s baseline status, age, thrombolysis status, and extent of area involved [1]. In this case, the patient’s baseline was poor, with a slow start of recovery, advanced age, and co-morbidities. Even when recovery is seen, patients often have complications, such as continued impaired memory and cognition [14].

## Conclusions

Bilateral thalamic strokes are rare due to the thalamus receiving independent branches from the posterior cerebral artery and posterior communicating artery. The artery of Percheron arises unilaterally from a single posterior cerebral artery (PCA) and supplies the thalami and anterior midbrain bilaterally. Occlusion of this artery can thus result in a bilateral thalamic stroke. The result is a coma-like presentation, where the patient will be difficult to rouse, with can delay diagnosis and impact the effectiveness of post-stroke therapy. A unique diagnostic challenge arises with such a stroke due to the low sensitivity of early imaging and the need for early thrombolysis. Understanding of its features and clinical judgment thus become of high importance. In emergency settings where the severity of clinical features, specifically the presence of significant impairment of consciousness, does not correlate with the initial imaging findings, one must perform follow-up imaging within therapeutic times to make the correct diagnosis when thrombolysis is still possible. Maintaining a high suspicion for thalamic infarct, with AOP occlusion as one etiology, is therefore crucial.
